# Conservative Management of a Rare Contiguously Spread Splenic Abscess

**DOI:** 10.7759/cureus.50747

**Published:** 2023-12-18

**Authors:** Arjun Chadha, Saakshi Joshi, Jaspreet K Ghumman

**Affiliations:** 1 Internal Medicine, McLaren Macomb Medical Center, Mount Clemens, USA; 2 Gastroenterology, McLaren Macomb Medical Center, Mount Clemens, USA

**Keywords:** splenic abscess, management of splenic abscess, pancreatic pseudocyst, pseudocyst, pancreatitis, abscess, splenic

## Abstract

Splenic abscess is a rare condition that generally results from hematogenous spread and affects individuals with hemoglobinopathies or immunocompromising conditions. Although optimal management has recently been under contention, this condition was traditionally managed with splenectomy. We present a rare case of a 58-year-old male with chronic pancreatitis that developed a splenic abscess via a contiguous spread of a pancreatic pseudocyst. His condition was complicated by septic shock. The splenic abscess was managed with antibiotics, image-guided percutaneous drainage, and notably without surgical intervention.

## Introduction

Splenic abscess is a rare condition, with autopsy series incidence ranging from 0.14 to 0.7% [1]. This condition typically results from hematogenous spread, often as a complication of bacterial endocarditis. Splenic abscesses have a predilection for patients with hemoglobinopathies or immunocompromised states. The optimal treatment modality is still controversial but includes the following options: acute surgical intervention, antimicrobial medical management, and percutaneous drainage [2].

In our case report, we describe a rare case of a splenic abscess resulting from the contiguous spread of a pancreatic pseudocyst in an immunocompetent individual. The condition was managed with antibiotic therapy and percutaneous drainage without the need for splenectomy.

## Case presentation

**Figure 1:**
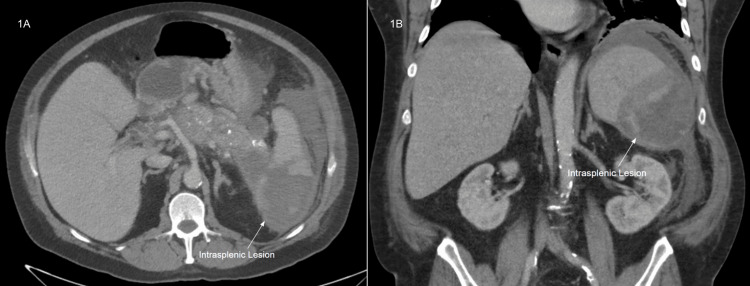
Intrasplenic lesion Transverse (A) and coronal (B) views of the pancreatic tail pseudocyst communicating with a large inferior intrasplenic abscess and additional extension into the superior spleen.

We report a case of a 56-year-old Caucasian male with a history of chronic pancreatitis secondary to alcohol use and complicated by pseudocyst, who presented to the emergency department (ED) with concerns about abdominal pain. Abdominal pain onset several days prior to admission and was characterized as a progressively worsening, colicky, sharp sensation localized to his left hypochondriac and flank regions. The patient also reported diaphoresis, chills, and nausea but denied fevers or emesis. The patient's condition in the ED was concerning for severe sepsis. Lipase was within normal limits (258 U/L). Computed tomography (CT) of the abdomen and pelvis with contrast identified acute on chronic pancreatitis with a pseudocyst developed near the pancreatic tail that communicated with a large intrasplenic abscess localized in the inferior spleen (Figure 1). 

Management was initiated per the sepsis protocol with septic bolus fluid resuscitation and broad-spectrum antibiotics with ampicillin-sulbactam. After inadequate hemodynamic response to fluid resuscitation, the patient was admitted to the intensive care unit (ICU) for septic shock, where he was initiated on Levophed® pressor support. The specialists from gastroenterology, infectious disease, general surgery, and interventional radiology were consulted. Infectious disease optimized the antibiotic regimen to meropenem and Diflucan®. General surgery identified the patient as a poor candidate for splenectomy, given his critical condition.

**Figure 2:**
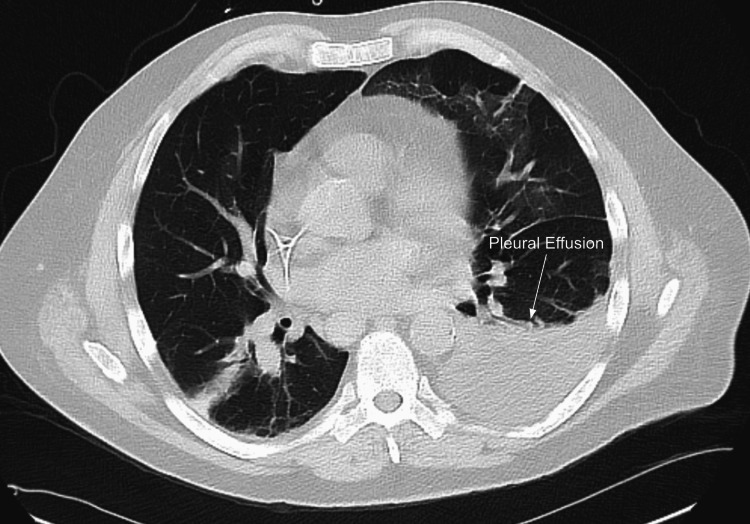
Moderate left-sided pleural effusion

On the second hospitalization day (HD), interventional radiology conducted an uncomplicated CT-guided percutaneous drainage of the splenic abscess and placed a 10-French drainage catheter. A transthoracic echocardiogram did not reveal vegetation concerning infective endocarditis. Blood cultures had resulted in alpha-hemolytic streptococcal bacteremia. On the third HD, ultrasound-guided paracentesis for loculated ascites was conducted with 1.9 L of dark, hemorrhagic fluid aspiration. Abscess and ascites fluid cultures both resulted in nonhemolytic streptococcus. On the seventh HD, a peripherally inserted central catheter (PICC) line was inserted for long-term antibiotic management. On the ninth HD, the antibiotic regimen was transitioned to ertapenem. On the 10th HD, CT for pleural effusion was conducted as the patient was dyspneic, and imaging revealed a moderate-sized left pleural effusion (see Figure 2). On the 13th HD, ultrasound-guided thoracentesis was conducted with 0.5 L of cloudy yellow fluid aspiration. On the 17th HD, the patient was discharged with the PICC line and intra-abdominal drainage catheter. The patient was instructed to continue ertapenem for three weeks outpatient via the PICC line. His intra-abdominal catheter was removed two weeks after his discharge, and his PICC line was removed upon the completion of the antibiotic course. The patient had marked improvement in symptoms upon completion of therapy.

## Discussion

Given the extensive vascularized nature of the spleen, splenic abscesses most often result from hematogenous spread [3]. Specifically, endocarditis has been identified as the leading cause of splenic abscesses. Other etiologies include splenic trauma, splenic infarction (notably in individuals with sickle cell disease), and gastrointestinal malignancies. Our case involves a rare presentation of a splenic abscess that resulted from the contiguous spread of a pancreatic pseudocyst. This rare pathophysiology of a splenic abscess is supported by CT evidence of communication between the pseudocyst and abscess.

During our evaluation of the patient, we considered the patient's diagnosis as a primary pancreatic pseudocyst that transformed into an abscess and simply extended and localized into the spleen. However, given that his hospitalization was complicated with streptococcal bacteremia, the species most often isolated in splenic abscesses [4], and pancreatic abscesses are more often associated with gram-negative isolates [5], we ultimately characterized this patient's pathology as a splenic abscess opposed to a pancreatic pseudocyst simply localized in the spleen. 

Our case is further unique given the immunocompetent nature of the patient. The common etiologies of splenic abscesses addressed earlier impact patients with underlying immunocompromising conditions and hemoglobinopathies. Neither status was identified in our patient.

Splenectomies have traditionally been considered the gold standard of treatment [6]. However, the recent advancement of image-guided percutaneous catheter drainage has facilitated a less invasive procedural approach. A recent meta-analysis comparing health outcomes between the two options revealed drainage as the superior option, given associations with lower complications and mortality rates [7]. Notably, these associations were not statistically significant, and the optimal treatment option for specific cases still relies on the patient's individual clinical scenario. Although our patient's management was further complicated with a superinfection requiring a paracentesis and thoracentesis, it was ultimately managed without a splenectomy. By salvaging the spleen, the patient will not be immunocompromised nor require lifetime antibiotic and vaccination management.

## Conclusions

We present a rare case of a contiguously spread splenic abscess in an immunocompetent individual. Splenic abscess is a rare condition that typically results from hematogenous spread and has been traditionally managed with splenectomy. In our patient, the splenic abscess resulted from the contiguous spread of a pancreatic pseudocyst and was successfully managed without surgical intervention.
